# Reply to Mammadov et al. Comment on “Catanzaro et al. Risk Factors for Recurrence of Primary Sclerosing Cholangitis after Liver Transplantation: Single-Center Data. *J. Pers. Med.* 2024, *14*, 222”

**DOI:** 10.3390/jpm16010055

**Published:** 2026-01-21

**Authors:** Elisa Catanzaro, Martina Gambato

**Affiliations:** 1 Department of Surgery, Oncology and Gastroenterology, University of Padova, 35128 Padova, Italy; 2 Gastroenterology Unit, Padova University Hospital, 35128 Padova, Italy; 3 Multivisceral Transplant Unit, Padova University Hospital, 35128 Padova, Italy

We thank Dr. Mammadov et al. [1] for their appreciation of our work [2]. We acknowledge their observations and the opportunity to clarify our findings.

**1.**  **Surgical Variables and Their Interpretability**

Regarding the use of Roux-en-Y anastomosis, we are aware of the potential indication bias behind the choice of surgical technique. As a retrospective study, only limited data were available, and we did not have detailed information on biliary anatomy. At the time of the study, we recognized that our results were in contrast with previous data, with two meta-analyses reporting no effect of reconstruction type on graft outcomes in PSC. However, the evidence specific to rPSC within these meta-analyses was limited. As highlighted in your comment, the meta-analysis by Wells et al. was used to frame the assessment of our study [3]. In this paper, the comparison of rPSC risk by anastomosis type derived from two studies, namely [4,5], that included a very limited number of events (six events in a pooled population of 66 patients), resulting in low-quality evidence. The statistical analyses in both papers were necessarily constrained by small sample sizes, as in our cohort. The median follow-up in Esfeh’s paper [4] was 36 months, whereas Heffron et al. [5] reported a follow-up more comparable with ours (duct-to-duct 5.1 ± 3.7 years and Roux-en-Y 4.1 ± 3.6 years, vs. 4.9 (IQR 3.7–6.0) years in our series. The shorter follow-up in Esfeh et al. could explain the limited number of observed events. More recently, the meta-analysis by Steenstraten et al. reported no clear impact of anastomosis type on rPSC (pooled HR 1.40, 95% CI 0.92–2.12), with a non-significant trend toward higher risk with Roux-en-Y [6]. This finding should be interpreted considering the marked imbalance between the groups (Roux-en-Y 1018 vs. duct-to-duct 182) and the historical nature of the cohorts (e.g., 1990–2006; 1984–2007), when rPSC definitions and surgical techniques differed from current practice. In our small cohort, only 4/33 patients underwent LT before 2000. Keeping in mind the limited size of our cohort and the potential for bias, we still attempted to offer a speculative biological hypothesis within a context where update evidence was lacking. To conclude with transparency, a recently published French cohort study provided new insight in the field, showing in a cohort of 571 transplanted PSC patients that the type of biliary anastomosis played no role in the development of rPSC, thereby filling the knowledge gap on this topic [7].

**2.**  **Graft Cold Storage Time**

We have to point out that rPSC likely does not reflect a single autoimmune pathway. Rather, given the multifactorial pathogenesis of the primary disease, recurrence probably arises from overlapping mechanisms. Donor-related characteristics such as longer CIT are related to extended-criteria donor (ECD) grafts and co-occur with other features (e.g., graft steatosis), already described as associated with recurrence in Alabraba et al. [8]. Given the small sample size of our cohort and limited power, these findings are hypothesis-generating only. They may suggest that, because of the additional risk of recurrence, PSC recipients could require more stringent graft selection than for other aetiologies; however, the underlying mechanistic pathway remains uncertain, and this interpretation remains speculative.

**3.**  **Unexpected Lack of Impact on Graft and Patient Survival**

We totally agree with your observation, given the extensive literature demonstrating effects on both patient and graft survival. In the Discussion, we clearly acknowledged this point as a limitation and noted that a plausible explanation is the small cohort size. We also fully agree that follow-up duration and post-transplant management at our center could have influenced the results. Finally, we cited one of the largest studies by Visseren et al. [9], reporting recent ELTR data—rather than relying solely on small or single-center series like ours—to openly highlight the controversy surrounding our finding.

**4.**  **Sample Size and Statistical Power**

Analogously with other studies included in the Wells et al. meta-analysis [3], we chose not to fit a multivariable model because of conventional events-per-variable considerations: with 33 patients and 9 events, a multivariable Cox model would be prone to overfitting and yield unstable estimates (inflated coefficients, wide confidence intervals). We therefore limited our analyses to univariable associations. We agree that this approach has limited statistical power (as noted above) and increases the risk of both type I error, given the multiple univariable comparisons, and type II error due to the very small number of events. Multicenter studies with larger cohorts could provide more robust, adjusted estimates. Nevertheless, this study represents the first Italian single-center experience reporting rPSC data; we considered it important to present contemporary data from an Italian transplant center and to facilitate comparison with other cohorts.

Regarding the additional risk factors (e.g., genotype or bloodstream infections), these variables were not fully available in the medical records, and, therefore, they could not be analyzed. However, the potential role of gut microbes in rPSC, as well as the potential impact of colectomy before transplant, has been widely investigated in the recent literature. Moreover, given the retrospective nature of data collection, it is difficult to ascertain whether patients tested for bacteremia were in a state of active infection at the time—introducing potential indication/surveillance bias—and the impossibility to standardize the timing between transplant and sampling could have represented another limiting aspect.

In conclusion, our analytic choices reflect the constraints of limited event counts and data availability. The findings were presented as exploratory, and at the time of writing a contemporary multicentric cohort assessment of some rPSC characteristics—such as the impact of the biliary anastomosis—was still lacking. Our aim was, therefore, to comment on and interpret the associations observed in our cohort.

With this in mind, we thank you for your commentary and appreciate the invaluable insights, which helped contextualize our study in relation to the existing literature in the field.
